# EDTNet: A spatial aware attention-based transformer for the pulmonary nodule segmentation

**DOI:** 10.1371/journal.pone.0311080

**Published:** 2024-11-15

**Authors:** Dhirendra Prasad Yadav, Bhisham Sharma, Julian L. Webber, Abolfazl Mehbodniya, Shivank Chauhan

**Affiliations:** 1 Department of Computer Engineering & Applications, G.L.A. University, Mathura, India; 2 Chitkara University Institute of Engineering and Technology, Centre for Research Impact & Outcome, Chitkara University, Rajpura, Punjab, India; 3 Department of Electronics and Communication Engineering, Kuwait College of Science and Technology (KCST), Doha Area, Kuwait; Khalifa University of Science and Technology, UNITED ARAB EMIRATES

## Abstract

Accurate segmentation of lung lesions in CT-scan images is essential to diagnose lung cancer. The challenges in lung nodule diagnosis arise due to their small size and diverse nature. We designed a transformer-based model EDTNet (Encoder Decoder Transformer Network) for PNS (Pulmonary Nodule Segmentation). Traditional CNN-based encoders and decoders are hindered by their inability to capture long-range spatial dependencies, leading to suboptimal performance in complex object segmentation tasks. To address the limitation, we leverage an enhanced spatial attention-based Vision Transformer (ViT) as an encoder and decoder in the EDTNet. The EDTNet integrates two successive transformer blocks, a patch-expanding layer, down-sampling layers, and up-sampling layers to improve segmentation capabilities. In addition, ESLA (Enhanced spatial aware local attention) and EGLA (Enhanced global aware local attention) blocks are added to provide attention to the spatial features. Furthermore, skip connections are introduced to facilitate symmetrical interaction between the corresponding encoder and decoder layer, enabling the retrieval of intricate details in the output. The EDTNet performance is compared with several models on DS1 and DS2, including Unet, ResUNet++, U-NET 3+, DeepLabV3+, SegNet, Trans-Unet, and Swin-UNet, demonstrates superior quantitative and visual results. On DS1, the EDTNet achieved 96.27%, 95.81%, 96.15% precision, IoU (Intersection over Union), and DSC (Sorensen–Dice coefficient). Moreover, the model has demonstrated sensitivity, IoU and SDC of 98.84%, 96.06% and 97.85% on DS2.

## 1. Introduction

Lung cancer remains to be the most prevalent and severe form of cancer on the globe. Lung cancer is defined by the proliferation of aberrant cells in the air passages of one or both lungs, resulting in uncontrolled growth [1]. Lung cancer is the primary cause of cancer-related fatalities worldwide, responsible for around 20% of all cancer deaths, as stated by the WHO (World Health Organization) [2]. The increased death rate happens due to the disease getting detected in an advanced stage, which poses more difficulties in achieving effective treatment.

Due to this, its impact on the patients, families, and health care system can be noticed through emotion and economics [3]. Unfortunately, at the initial stage of cancer, patients have fewer symptoms of the disease. Hence, an automated system that can detect cancer at the stage of disease needs to be developed [4]. With the advancement of technology, several image processing-based techniques have been developed that can be used for the diagnosis of lung cancer. Computed tomography (CT) imaging is one of the imaging tools used for lung cancer screening. It provides comprehensive details of the cross-sectional body view. Another image processing technique is X-ray imaging, which includes body details in different black-and-white shades [5]. X-ray processing is fast but cannot detect smaller lesion regions of the lung cancer for high-risk lung cancer patients. LDCT (Low-dose CT) is utilized because it can reduce lung cancer, such as in smokers and older individuals, by facilitating the identification of cancer at a more manageable stage [6]. However, screening of nodules with oval-shaped lung growths and small size remains challenging. Due to this, it is difficult to differentiate between benign and malignant nodules in cancer patients. Therefore, a precise and robust system is required for the segmentation of the small size nodule. In addition, manual screening of the CT-scan image is time-consuming and subject to variability in interpretations among radiologists [7,8].

Early efforts in pulmonary nodule detection involved manual examination and basic image processing. Integrating computer-aided detection (CAD) systems marked a significant advancement, initially relying on rule-based algorithms and later incorporating more advanced machine-learning techniques for improved detection accuracy [9]. However, using machine learning with algorithms like SVM (Support Vector Machines) and Random Forests, relying on handcrafted features [10]. The medical imaging landscape has been transformed with convolutional neural networks (CNNs). CNNs learn feature representations directly from data, eliminating the need for manual feature extraction. This development substantially enhanced the accuracy and efficiency of nodule detection. Deep learning models consistently outperformed traditional CAD systems, as demonstrated in a landmark study that showcased their ability to achieve higher sensitivity and specificity, occasionally surpassing human radiologists [11,12]. Moreover, the introduction of 3D CNNs, which utilize the volumetric information of CT scans, has been a further enhancement. These models provide a comprehensive analysis and have shown promise in detecting smaller, subtler nodules [13]. Recently, several studies applied the ViT (Vision transformer) model to segment lung cancer in CT images precisely [14,15].

In short, the inspection of lung nodules in clinical practice is time-consuming and requires expert radiologists to diagnose the disease. The evolution of image processing and computer vision improved the diagnosis process through automated systems. However, machine learning-based methods are prone to error due to manual hand-crafted features utilized for the training of the model. Moreover, the CNN-based method improved the medical image diagnosis task through automatic feature extraction capability. However, the sallow high dimensional spatial features extracted by the CNN are less effective in several applications. Recently, ViT has emerged as a powerful method which provides long-range dependency on spatial features to improve classification accuracy. At the same time, designing a lightweight ViT model is a complex task.

The 3D image processing is computationally extensive compared to 2D since we require a 3D convolution neural network to analyze the dataset. In addition, annotation of the 2D slices from the CT scan is easier and faster. Furthermore, radiologists often examine the disease with 2D slices in clinical practice. Therefore, we utilized two-dimensional images rather than three-dimensional images for the segmentation of pulmonary nodules.

This study presents the EDTNet, a transformer-based model for PNS. Conventional CNN-based encoders and decoders face limitations in capturing long-range spatial dependencies, affecting their efficacy in complex object segmentation. To overcome this, the EDTNet employs an enhanced spatial attention-based Vision Transformer (ViT) as both encoder and decoder. The architecture integrates two successive transformer blocks, downsampling layers, upsampling layers, and a patch-expanding layer, enhancing its segmentation capabilities. A strategically designed bottleneck featuring an up-sampling layer, a down-sampling layer, and a bottleneck block consisting of three global transformer blocks are incorporated to refine the decoder’s functionality. Furthermore, skip connections foster symmetrical interaction between the corresponding encoder and decoder layers, facilitating the retrieval of intricate details in the output. Further, we compared performance with several models, including Unet, ResUNet++, U-NET 3+, DeepLabV3+, SegNet, Trans-Unet, Swin-UNet, HTC-Net, VM-UNet and achieved better quantitative and visual results.

The proposed method makes a noteworthy contribution in the following ways:

- We designed a transformer-based encoder and decoder, which effectively addresses the challenge of capturing extensive spatial feature dependencies and enhancing performance in intricate segmentation tasks.
- To boost the segmentation capabilities of the model, the EDTNet integrates two consecutive transformer blocks involving downsampling, upsampling, and a patch-expanding layer. In addition, a cross-attention module in the decoder is implemented to enhance the contextual information.
- We incorporated the attention module ESLA (Spatially Aware Local Attention) in the encoder and decoder block and ESGA (Spatially Aware Global Attention) in the bottleneck. These two attention modules are tailored to capture details of edges and small-sized lung nodules.
- The superiority of the EDTNet is evaluated on the three open source datasets and compared with several methods via visual and quantitative results.

The rest of the paper is organised as follows.

In section 2, we provide an extensive analysis of the previous studies, whereas in section 3, we present the architecture of the proposed model. In addition, section 4 provides a detailed analysis of the quantitative and visual outcomes achieved by the proposed approach. In section 5, we eventually summarize the suggested method.

## 2. Related work

In this section, we present the methods utilized for the PNS using computer vision-based techniques. Rocha et al. [16] compared the traditional and deep learning (DL) approaches for PNS in CT scan images. The conventional approach uses the SBF (sliding band filter) to assess support points in the border coordinate. The same segmentation process was conducted using U-Net and SegU-Net. The U-Net achieved the highest precision of 89.8% on the nodules dataset. Lu et al. [17] introduce a U-Net-based network for PNS in CT images. They utilized dense connections to enhance feature transfer and prevent gradient disappearance, as well as a new loss function that considers pixels near nodule borders to improve performance. Balcı et al. [18] proposed a hybrid approach for classification. They perform a radial scan to obtain ROI (Region of interest) from images. After that, they use a U-shape CNN for 4D data classification in lung nodule images.

Cao et al. [19] introduce the DB-ResNet (Dual-branch Residual Network) for lung image segmentation. Firstly, the model enhances generalization by capturing multi-scale features and multi-viewing nodules in CT images. After that, they combine intensity and CNN features using a CIP (central intensity pooling layer). Finally, a weighted sampling strategy was utilized to enhance accuracy. Chen et al. [20] compared the performance of several DL models, including SegNet, UNet, FCN, GCN, PspNet, DeepLabV3+, SwinUNet and TransUNet for lung nodule segmentation. They explored the impact of various pre-processing methods. Including the nodule data from the ROI without a lung mask leads to better segmentation results.

Zhang et al. [21] utilized a DL method for efficient lung nodule identification using the MSDLF (Multi-Scene Deep Learning Framework) with a vesselness filter. The MSDLF was trained on the 3D images to enhance the detection of nodules by combining two images of different datasets. This framework improves accuracy and reduces false positives in detecting lung nodules within extensive image datasets. Khan et al. [22] introduce the VGG-SegNet framework for nodule mining and a pre-trained DL-based method for classification. In addition, they employed deep features with handcrafted features to improve disease detection accuracy. Maqsood et al. [23] utilized a U-Net-based model. Their model, DA-Net, has a similar end-to-end framework for precise segmentation. They combine Atrous convolutional blocks and dense convolutional blocks to capture extensive features without compromising data. Initially, they calculate the standard deviation and k-means clustering algorithm to extract the ROI, focusing the model on the relevant area.

Bhattacharjee et al. [24] suggested a segmentation algorithm that leverages a dual skip connection-based approach. The ResiU-Net integrates a fine-tuned ResNet152 and the U-Net architecture through rigorous analysis of nine pre-trained and fine-tuned encoder models. They discovered that the ResiU-Net approach outperformed those without fine-tuned ResiU-Net. In another study, Roy et al. [25] developed an automated lung nodule segmentation framework integrating deep learning with shape-driven level sets. This approach uses a coarse-to-fine strategy, with initial segmentation performed by a deep convolutional network. Fine segmentation is achieved through the evolution of level sets, utilizing seed points derived from the deep CNN.

Yu et al. [26] designed a 3DRes U-Net network for segmentation and a 3D ResNet50 for classification using 3D CT images. Their method improves the traditional U-Net by incorporating residual units in its convolutional layers and adopts dice loss alongside cross-entropy loss for improved network convergence. Furthermore, to better capture the 3D characteristics of lung nodules, the 2D convolutional layers in ResNet50 are replaced with 3D convolutional layers, resulting in a 3D ResNet50 network for diagnosing benign and malignant lung nodules. Aresta et al. [27] introduced a deep CNN model named iW-Net for lung nodule segmentation. They allowed users to make corrections interactively by marking two points on the nodule’s boundary.

Chiu et al. [28] designed a 2D U-Net model to identify lung nodules. They address the challenge of imbalanced labelling in medical images where lung nodules are relatively rare in the overall image. The study employs the dice coefficient loss for evaluation and a pre-processing technique that involves swapping labels, designating the nodule position as non-labelled and the no-nodule position as labelled. The pyramidal attention-based model was developed by Bruntha et al. [29] for enhancing lung nodule segmentation in low-dose CT images. They utilized an inverted residual block, swish activation, and a feature pyramid attention network for precise feature extraction. Compared to the conventional UNet, Lung_PAYNet demonstrated significant improvements in results.

Ciompi et al. [30] applied a DL model for classifying nodule types. The model processes raw CT data and learns 3D data by analysing 2D views of a lung nodule. They trained the model on Italian Multicentre Italian Lung Detection (MILD) data and validated it on DLCST (Danish Lung Cancer Screening Trial) data. The system outperforms classical machine learning approaches. Wang et al. [31] propose a DL model that combines segmentation and classification to improve the diagnosis of pulmonary GGNs (ground-glass lung nodules). The segmentation model generates an attention map that guides the classification model, leading to better performance.

Several CNN and transformer based model have been used for medical image segmentation. Unet [32], ResUNet++ [33], U-NET 3+ [34], DeepLabV3+ [35], SegNet [36], Trans-Unet [37], Swin-UNet [38], HCT-Net [39] and VM-UNet [40]. The U-Net is a CNN network designed for image segmentation. It has a distinctive U-shaped architecture with a contracted channel for downsampling and an expanded path for upsampling. The encoder is typical of a CNN structure, with recurrent convolution and max pooling operations that double the feature channels with each step. On the other hand, the decoder incorporates upsampling and convolution, halves the feature channels, and is supplemented by skip connections from the contracting path. ResUNet++ is an advanced adaptation of the U-Net architecture. It utilized residual connections to mitigate the vanishing gradient problem, enabling the construction of deeper networks without losing training efficiency. In addition, dilated convolutions are incorporated to expand the receptive field of the network, allowing it to capture broader contextual information without an increase in parameters.

Furthermore, an attention mechanism was provided using squeeze-and-excitation blocks to focus more effectively on relevant features by modelling interdependencies between channels. The U-Net 3+ model is specifically crafted to enhance performance in medical image segmentation tasks, building upon the foundational U-Net structure with noteworthy improvements. It introduces full-scale skip connections, a refinement over the conventional skip connections in U-Net. These connections are strategically designed to integrate features from various scales more effectively, enabling the network to capture high-level semantic information and low-level details more efficiently. Furthermore, U-Net 3+ incorporates deep supervision mechanisms to fine-tune segmentation accuracy across different resolution scales.

DeepLabV3+ controls the features resolution response with CNN through atrous convolution, which helps capture the multiscale spatial information. The atrous convolutions of different sizes in the ASPP (Atrous Spatial Pyramid Pooling) module improve the segmentation accuracy. In addition, it is adequate to capture image features and fine details. The SegNet is an encoder and decoder-based model having several convolution layers followed by a BN (Batch normalization) and ReLU (Rectified Linear Unit) activation function. Furthermore, the spatial dimension is reduced using a max-pooling layer to capture the contextual information and distinctive features within the image. Subsequently, it preserves the highest feature value for the upsampling process. The decoder block uses the pooling indices to generate a features map, which is crucial for accurately reconstructing the image to its original resolution. The HTC-Net is a hybrid model designed using CNN and transformer. Whereas, the VM-UNet is designed using Visual State Space (VSS) for contextual feature extraction.

## 3. Proposed method

### 3.1. Datasets

The DS1 dataset includes 40 patients exhibiting malignant features and 561 images depicting patients with malignancies. Out of these, 204 images were selected, and corresponding ground truth (GT) was generated with the guidance of professionals SMCH (from Silchar Medical College and Hospital) Assam, India. The images of cancerous nodules were divided into distinct segments that can be associated with ground truths (GTs). Furthermore, images were cropped to highlight the ROI. Subsequently, images were resized to 256x256 pixels. The deep learning model requires extensive data for training to achieve better generalization. In the DS1, not many training samples are available, potentially influencing the model’s performance. Hence, various data augmentation approaches were applied, such as horizontal and vertical flips, 90° and 180° rotations, and additional cropping. Finally, DS1 has 1224 images for training and validation of the model [24]. **Last access 4/April/2023.**

The DS2 is collected from the Decathlon challenge (http://medicaldecathlon.com/), which contains data on various segmentation tasks. Track06 contains images of lung nodule segmentation. It has small lung cancer images of 96 patients with their segmented masks for GT. We selected 96 CT-scan slides, and 1657 images with their GT were generated for the lung nodule segmentation. **Last accessed 7/September/2023.**

**Fig 1:**
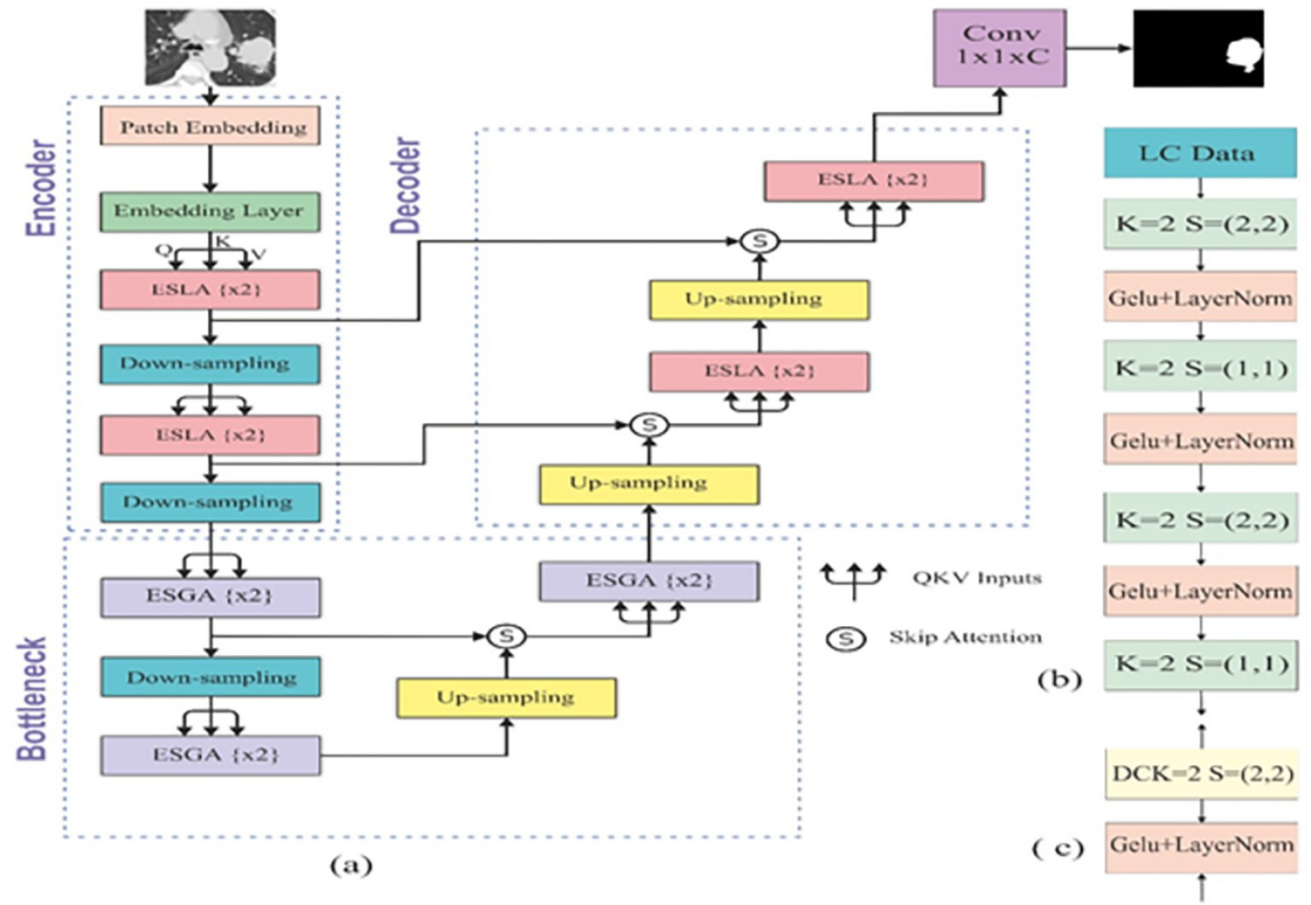
The architecture of EDTNet. In (a) the overall architecture of the EDTNet, (b) details of the embedding layers and (c) the upsampling process. Where S, K, DCK, represents stride, Kernel and deconvolution kernel.

In the proposed study, we developed an EDTNet inspired by UNet. The UNet has CNN as an encoder and decoder. The CNN-based encoder and decoder structure ignore the long-range dependency of the spatial features [41,42]. Due to this, performance is lower in several complex scenarios [43]. The segmenter model follows the transformer as encoder and decoder to improve the segmentation of the objects [44]. The EDTNet utilized an enhanced attention-based ViT as an encoder and decoder for the PNS. We used two successive transformer blocks and two downsampling layers in the encoder. Similarly, our decoder contains two upsampling layers, two transformer blocks and one patch-expanding layer. The bottleneck of the architecture also includes a downsampling block, an upsampling block, and three global transformer blocks. Each component expands the receptive field, improving the decoder’s functionality. Our model is inspired by the U-Net architecture [45]. We utilized skip connections among the encoder and decoder’s associated layers. This symmetrical arrangement facilitates the restoration of intricate details in the output. Contrary to the classical approach of using summation or concatenation in skip connections, our design employs a unique skip attention mechanism. The skip attention effectively links the encoder and decoder, enhancing the information flow and feature integration between these critical model components. The structure of the suggested model is depicted in Fig 1.

Let $I\in R^{H\times W\times C}$ be the input image divided into overlapping patches P_i_. We extracted spatial features using convolutional operations as follows.


$$F_{i}=Conv(P_{i})$$ (1)

Each patch feature, F_i_, is then linearly transformed into a higher-dimensional space as follows.

$$E_{i}=F_{i}W_{i}$$ (2)

where W_E_ is the learned weight matrix. After that, enhanced positional encodings(EPE) is applied for the patch embedding using Eq (3).

$$E_{i}^{\prime }=E_{i}+PE_{i}$$ (3)

Where PEi are learnable parameters. For ESLA (spatially aware local attention), we calculated the Q (query), K (key) and V (value) as follows.


$$Q=E^{\prime }W^{Q},K=E^{\prime }W^{K},V=E^{\prime }W^{V}$$ (4)

The SA (self-attention) module calculates attention on the images through the Q, K, and V. The pulmonary nodule images have complex affected regions. Therefore, we applied the spatial bias that includes learnable parameters focusing on small and complex regions. In addition, spatial bias allows the model to adjust the bias dynamically. Furthermore, learnable weights W_S_ are optimized during training, and spatial positions of patches are encoded as vectors P_i_ and P_j_ (positions of patches i and j, respectively). Finally, the spatial bias is calculated as follows.


$$S_{ij}=h(P_{i},P_{j},W_{s})$$ (5)

Here, h is a function that combines the position vectors and the learnable weights to compute the bias. After computation, the spatial bias is integrated into the ESLA mechanism, and modified attention scores with spatial bias are calculated as follows.

$$Attention(A)=Soft\max(\frac{QK^{T}}{\sqrt{D_{k}}}+S)V$$ (6)

**Fig 2:**
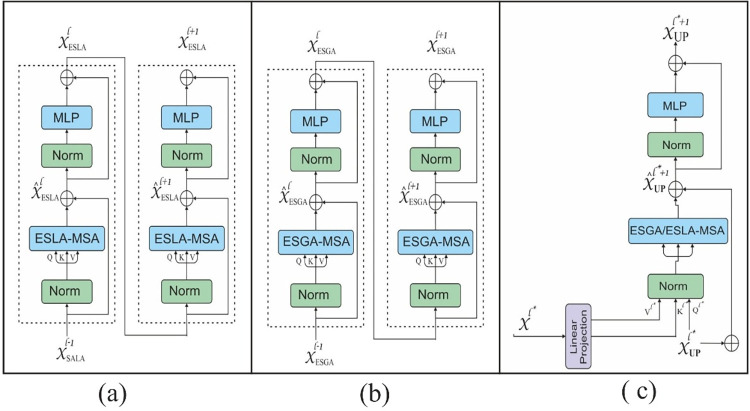
The architecture of the ESLA-MHA, ESGA-MHA, and CA attention is shown in (a), (b), and (c), respectively.

Where S represents a spatial bias term that encodes the relative positions of patches, the Q, K, and V are the query, key, and value matrices. The QK^T^ computes the standard attention scores, and S is the spatial bias matrix, added to the attention scores before applying the softmax function. After that, the softmax function normalizes these scores and creates a weighted sum of the values. These values are passed to the bottleneck block of the proposed model. The bottleneck module has a transformer block that contains ESGA (enhanced spatially aware global attention) with a similar structure to the blocks in the encoder but is tuned to process the deepest level of features. It compresses the high-dimensional features extracted by the encoder into a more compact and essential representation. Further, we reduce the dimensionality of the feature space. This reduction is not just in terms of spatial dimensions but also in the feature space itself, ensuring that only the most crucial information is passed to the decoder. The ESLA-MHA (enhanced spatial aware MHA), ESGA-MHA (enhanced spatial aware global attention MHA), and CA (cross attention) of the proposed method are shown in Fig 2.

### 3.2. Decoder with enhanced cross-attention

The design of the two transformer blocks in the decoder and encoder is highly symmetrical. However, unlike the encoder’s down-sampling blocks, the decoder uses stride deconvolution to elevate the feature maps from a lower to a higher resolution. Skip attention integrates these up-sampled feature maps with the encoder’s outputs. This approach effectively combines semantic context and detailed information. Correspondingly, the final stage of the up-sampling process also involves a deconvolution operation performed by the last patch-expanding block, which is responsible for generating the final mask predictions. Let D be the decoder input, and then upsampling can be represented as follows.


$$D^{\prime }=UpSample(D)$$ (7)

In the cross-attention layer, decoder embedding attends to encoder outputs as follows.


$$D^{\prime \prime }=D^{\prime }W^{\prime }$$ (8)

The output E_enc_ computed from the encoder block is passed to the decoder block. The cross-attention of the decoder is calculated as follows.


$$C=Soft\;\max(\frac{D^{\prime \prime }E_{enc}^{T}}{\sqrt{D_{k}}})$$ (9)

We added enhanced skip connections to attain the attention-based fusion of encoder and decoder features as follows.


$$S_{i}=Attention(D_{i},E_{enc,i})$$ (10)

Further, the positional encodings to the decoder inputs are provided as follows.

$$D_{final}=D^{\prime }+PE_{dec}$$ (11)

Where PE_dec_ are the positional encodings for the decoder of the EDTNet.

### 3.3. Training and loss function

The training loss is important to evaluate the model’s performance. High training loss leads to a less reliable model. In the proposed study, we designed the loss function for segmentation by combining the Dice Loss and Cross-Entropy Loss. The hybrid loss function is defined as follows.

$$L_{h}=\alpha \times DiceLoss(Y,Y^{\prime })+(1-\alpha )CrossEntropy(Y,Y^{\prime })$$ (12)

Where Y is the ground truth, Y^’^ is the predicted segmentation, and α is a balancing parameter. The algorithm of the proposed method is shown below.

**Algorithm 1. The proposed method for PNS.**

**Input:** Image and ground truth

**Output:** Segmented image

**Require:** Adam optimizer, batch size, epochs, initial learning rate

**Initialize:** opt = ‘Adam’, batch_size = 64, epcohs = 200, learning_rate = 0.0001

(1) Resize input image to 256x256x3 pixels

 (2) Split the dataset randomly into 80% and 20 % for training and validation

(2) Perform convolution embedding using Eq (1)

(3) Generate Q, K and V using Eq (4)

(4) Generate features from Q, K and V passing to the encoder block

(5) for i = 1 to 200 do

          (a) Generate the performance measures

           (b) Plot the training loss

       end

 (6) Predict the segmented image.

## 4. Experimental results

In this section, we will discuss the quantitative and visual results of the EDTNet on DS1.

### 4.1. Experimental setup

We conducted experiments using an NVIDIA Quadro RTX 4000 GPU. It has 128 GB RAM and a Windows 10 system equipped with two graphics cards, each with 8GB of memory. The experiments’ scripting was written using Python 3.9 and TensorFlow 2.1. The training process was accelerated using an Adam optimizer with an initial learning rate of 0.0001. Furthermore, in each experiment, images with dimensions of 256x256x3 pixels were used as input for training the model in a batch size of 64 for 200 epochs.

### 4.2. Performance evaluation parameters

The SDTNet performance was evaluated through indicators precision, sensitivity, IOU (Intersection Over Union), and SDC (Sorensen Dice coefficient)

**4.2.1. Precision**. It quantifies the level of accuracy of positive estimations. Mathematically, it is calculated by dividing the number of correctly identified positive cases by the total number of positive cases predicted.


$$\mathrm{Precision}=\frac{TP}{TP+FP}$$ (13)

TP and FP are the number of true positives and false positives samples.

**4.2.2. Sensitivity.** The metric quantifies the accuracy of identifying positive instances by calculating the ratio of correctly estimated true positives to the total number of positive instances. The mathematical expression for sensitivity is as follows.

$$\mathrm{Sensitivity}=\frac{TP}{TP+FP}$$ (14)

Where TP and FN are the number of true positives and false negatives.

**4.2.3. Intersection over Union (IoU).** IoU serves as a quantitative metric for evaluating the accuracy of a target detector. It is calculated through the intersection and union of the predicted and ground truth sets.

$$IoU=\frac{\left|A\cap B\right|}{\left|A\cup B\right|}$$ (15)

Where A and B are the predicted segmentation and the ground truth, respectively.

**4.2.4. The Sorensen-Dice coefficient (SDC).** It is a measure of the degree of overlap between two samples, providing a quantitative assessment of the similarity between two sets [46]. Mathematically, the SDC is defined as follows.


$$\mathrm{Dice}=2\times \frac{\left|A\cap B\right|}{\left|A\cup B\right|}$$ (16)

In the binary segmentation, A and B are the predicted and the ground truth set of pixels, respectively.

### 4.3. Quantitative results

In Table 1, we summarize the performance of several methods for PNS on different datasets under different experimental setup. Bhattacharjee et al. [24] designed ResiUnet using ResNet152 achieved 95.02% IoU. Further, Tang et al. [47] utilized multi-scale features fusion model SM-RNet (scale-aware-based multi-attention-guided reverse network) for PNS. The. Li et al. [48] segmented the pulmonary nodule using MRBU-Net-WD. The MRBU-Net-WD is a residual 3D convolution-based model. In addition, a Bi-FPN module is added to enhance feature fusion. This model achieved a 96.63% IoU value on the LUNA-16 dataset. Wang et al. [49] utilized BorDenNet to segment lung nodules on the LIDC–IDRI dataset. In another research, Lin et al. [50] designed a 3D network using Inception and Residual blocks and performed experiments on the LUNA16 dataset. Usman et al. [51] segmented the lung nodule using DEHA-Net (dual-encoder-based hard attention network). The DEHA-Net achieved an IoU value of 90.84% on the LIDC/IDRI dataset. Rani et al. [52] utilized AMPWSVM (Advanced Marine Predator algorithm with SVM) for lung tumour detection. The small region nodule segmentation is carried out by Albert et al. [53] using the WSBTI (watershed segmentation-based topological interpretation) method. Their method achieved 97% IoU, the highest in the Table. However, the dataset size is much larger than the other methods.

**Table 1:** Performance comparison on different datasets.

Study	Images	Model	IoU
Bhattacharjee et al. [24]	1224 CT-Scan	ResNet152	95.02%
Tang et al. [38]	888 CT-Scan	SM-RNet	89.20%
Li et al. [48]	888 CT-Scan	MRBU-Net-WD	96.63%
Wang et al. [49]	27583 CT-Scan	BorDenNet	92.78%
Lin et al. [50]	888 CT-Scan	IR-UNet + +	95.78%
Usman et al. [51]	244527 CT-Scan	CADe	90.84%
Rani et al. [52]	244527 CT-Scan	AMPWSVM	93.30%
Albert et al. [53]	244527 CT-Scan	WSBTI	97.00%
Tang et al. [54]	1134 CT-Scan	Res2Net50	90.07%
Li et al. [55]	888 CT-Scan	REMU-Net	84.76%
Proposed method	1224 CT-Scan	EDTNet	95.81%

Tang et al. [54] proposed a UNet-based network considering Res2Net50 as a backbone for nodule segmentation. Their method facilitates lung nodule segmentation with 90.07% IOU on the 1134 CT-scan images. Furthermore, Li et al. [55] proposed a REMU-net and achieved 84.76% IOU on the 888 CT-scan images.

For fair comparison, we implemented Unet [32], ResUNet++ [33], U-NET 3+ [34], DeepLabV3+ [35], SegNet [36], Trans-Unet [37], Swin-UNet [38], HTC-Net [39], VM-UNet [40] and the proposed EDTNet on DS1 under same experimental setup. The DS1 images were randomly split into 80% and 20% for training and validation, respectively. After that, images of size 256x256x3 are fed to the model for training and validation. Then model was trained for 200 epochs in a batch size of 64 using the Adam optimizer and initial learning rate for the optimizer was set to 0.0001. The performance measures precision, sensitivity, IoU and SDC of the different methods on DS1 are shown in Table 2.

**Table 2:** Performance of the SEDTNet on DS1.

Model	Precision (%)	Sensitivity (%)	IoU(%)	SDC (%)
Unet [41]	82.14	85.17	81.37	82.05
ResUNet++ [42]	85.27	84.89	83.47	84.17
U-NET 3+ [43]	81.52	80.34	80.15	80.94
DeepLabV3+[44]	83.68	82.19	81.59	82.72
SegNet [45]	78.86	80.31	79.23	77.82
Trans-Unet [46]	92.18	93.14	91.85	92.04
Swin-UNet [32]	94.28	95.61	92.32	93.56
HTC-Net [39]	93.86	94.37	92.15	93.16
VM-UNet [40]	94.17	93.72	91.68	92.57
Proposed EDTNet	**96.27**	**98.34**	**95.81**	**96.15**

Table 2 shows that the SegNet achieved the lowest precision and sensitivity values of 78.86% and 80.51%, respectively. The U-NET 3+ obtained 81.52% and 80.34% precision and sensitivity values. Furthermore, the Unet and DeepLabV3+ have IoU values of 81.37% and 81.59% respectively. The ViT-based model Trans-Unet and Swin-UNet achieved a DSC value of 92.04% and 93.56% respectively. The HTC-Net and VM-UNet achieved better IoU value compared to tradition CNN based encoder and decoder. Meanwhile, the proposed EDTNet achieved the highest precision and IoU values of 96.27% and 95.81%, respectively.

In Table 3, we depicted the performance measures of the Unet [32], ResUNet++ [33], U-NET 3+ [34], DeepLabV3+ [35], SegNet [36], Trans-Unet [37], Swin-UNet [38] and the proposed EDTNet on the DS2. We can see that the IoU value of the classical CNN based models are relative less compared to the ViT based models. The SegNet has precision 80.56%, least in the table. Whereas proposed EDTNet and Swin-UNet has 97.67% and 98.31% precision value. The Swin-UNet utilized transformer in the encoder and decoder, which provide global co-relation of the spatial features. Due to this some performance indictor value is slightly high on the DS2. The HTC-Net and VM-UNet achieved 94.80% and 95.06% SDC on DS2. In addition, we can notice that SDC value of the transformer based methods are relative high compared to traditional CNN based model and highest value of 97.85% achieved by the EDTNet.

**Table 3:** Performance of the SDTNet on DS2.

Model	Precision (%)	Sensitivity (%)	IoU(%)	SDC (%)
Unet [41]	85.43	82.57	83.94	84.25
ResUNet++ [42]	83.48	81.67	81.14	82.16
U-NET 3+ [43]	89.67	85.21	84.52	82.75
DeepLabV3+[44]	93.05	91.27	90.73	91.13
SegNet [45]	80.56	78.49	77.98	76.86
Trans-Unet [46]	94.42	95.34	93.48	94.18
Swin-UNet [32]	**98.31**	97.85	95.10	95.12
HTC-Net [39]	95.32	94.72	94.37	94.80
VM-UNet [40]	96.29	95.36	95.23	95.06
Proposed EDTNet	97.67	**98.84**	**96.06**	**97.85**

### 4.4. The qualitative results

**Fig 3:**
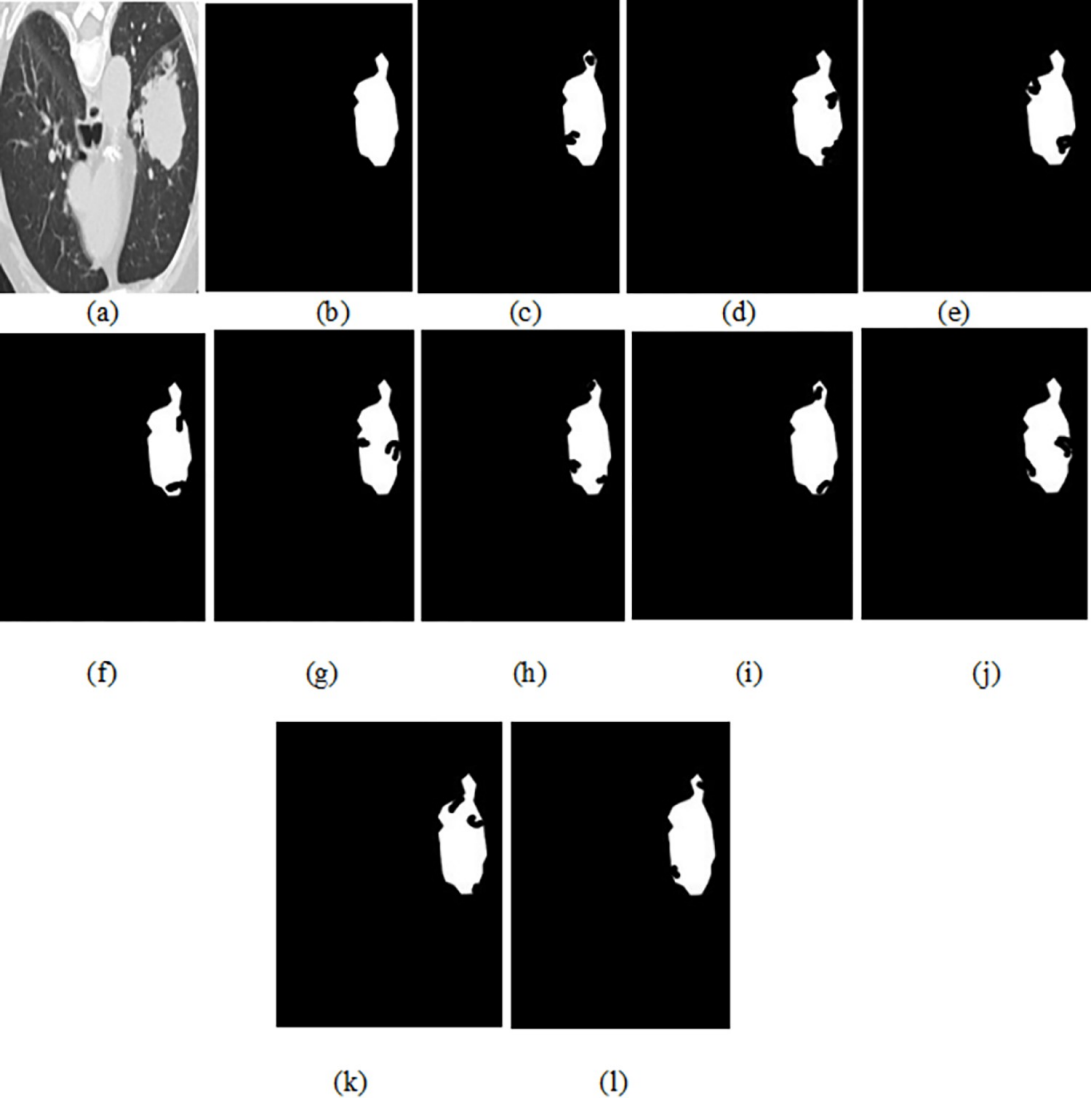
The predicted Figure, (a) original image, (b) GT (c)Unet (d) ResUNet++ (e) U-NET3+ (f) DeepLabV3+ (g)SegNet (h)Trans-Unet (i) Swin-UNet (j)HTC-Net, (k)VM-UNet and (l) EDTNet.

**Fig 4:**
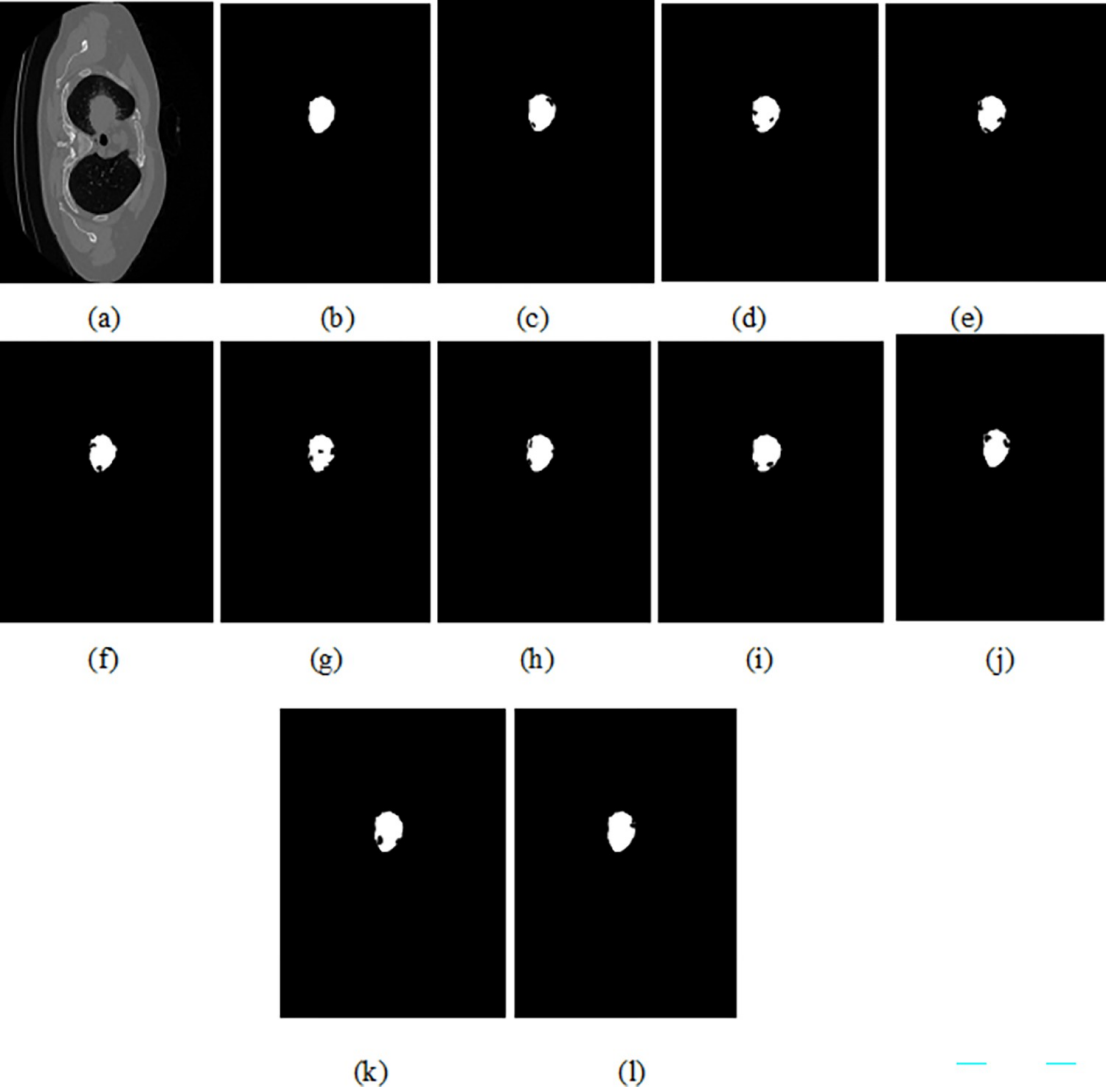
The predicted Figure, a) original image, (b) GT (c)Unet (d) ResUNet++ (e) U-NET3+ (f) DeepLabV3+ (g)SegNet (h)Trans-Unet (i) Swin-UNet (j)HTC-Net, (k)VM-UNet and (l) EDTNet.

The original image (top), GT (ground truth) and predicted image of the Unet, ResUNet++, U-NET 3+, DeepLabV3+, SegNet, Trans-Unet, Swin-UNet and the proposed EDTNet is shown in Figs 3 and 4. We can notice that the Unet segmented image has good boundary delineation of lung nodules. Its effective encoding-decoding structure that captures context and details produces high accuracy. However, they missed some finer details or smaller nodules due to the limitations in capturing very deep features. The improved segmented image can be seen in ResUNet++. The ResUNet ++ residual connections help preserve edge information. However, noise is available where highly irregular or very small nodules are present in the original image. The U-Net 3+ produced superior segmentation with enhanced details, as this model integrates multi-scale features effectively. It captured better at various nodule sizes and shapes due to its deeper and more comprehensive feature integration. The complex structure of the model requires more computational resources.

Meanwhile, DeepLabV3+ generated high-quality segmentation with excellent boundary adherence, utilizing atrous convolutions for multi-scale context. However, it fails to generate accurate masks on the edges. In addition, it requires longer inference times. The SegNet visual map has noise on several edge and boundary regions. This model is effective for larger nodules, emphasizing visual clarity in the segmented output. In addition, SegNet is not capable of capturing excellent details as effectively as compared to other models. Much finer visual results are produced by the Trans-UNet. The Trans-UNet is effective in capturing both global and local features, potentially leading to superior segmentation of nodules. The complexity of the model might result in more training and inference times. The Swin-UNet also resulted in finer masked images. Since it focuses on the hierarchical representation of the spatial features, it fails to handle diverse nodule sizes and complex lung backgrounds, capturing detailed and abstract features efficiently. In addition, it requires substantial computational resources due to its sophisticated architecture. The HTC-Net and VM-UNet visual map has less noise compared to the tradition CNN based encoder and decoder models. The EDTNet obtained a similar masked image compared to GT. In EDTNet, we designed the ESLA block to preserve the local spatial information of the edges and boundary region. In addition, we utilized the ESGA block to preserve the information obtained from the encoder to the bottleneck layer.

### 4.5. The training loss

**Fig 5:**
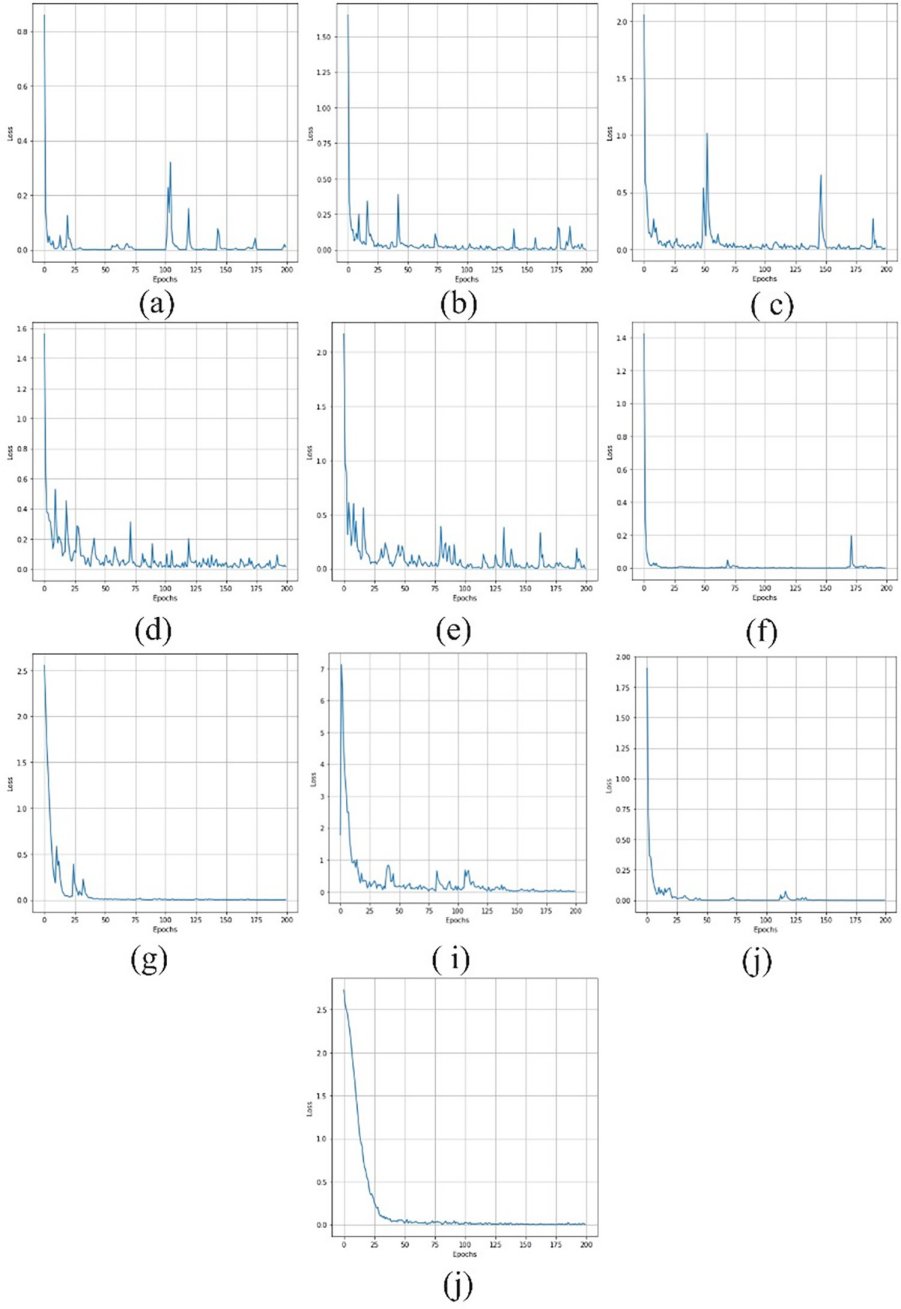
The training loss of the (a) Unet, (b) ResUNet++ (c) U-NET 3+ (d) DeepLabV3+ (e) SegNet (f) Trans-Unet (g) Swin-UNet (h) HTC-Net (i) VM-UNet (j) EDTNet.

The training loss curve provides a visual representation of how the model’s error decreases over epochs. A steadily decreasing loss curve indicates that the model is learning and converging towards optimal parameters. Comparing loss curves of different models helps in selecting the best-performing model [56,57]. The training loss curves for various models, including Unet, ResUNet++, U-NET 3+, DeepLabV3+, SegNet, Trans-Unet, Swin-UNet, and proposed EDTNet, are illustrated in Fig 5. Examining these curves, we observe distinct patterns for each model. For Unet, the curve is characterized by numerous high and low fluctuations, indicating variability in its training process. ResUNet++ displays a different trend, with initially high loss values that significantly diminish, approaching near zero after 195 epochs. This confirms that the model is gradually improving training performance over many epochs. In the U-NET 3+, we can notice high and lock peaks at the beginning of the training, and they started decreasing after 150 epochs, indicating better stabilization of the training [58].

Meanwhile, DeepLabV3+ has a better training curve from the beginning of the training. It has some high peaks with a loss close to 0.2 after 100 epochs, indicating a consistent and stable training process. However, SegNet has several high and low peaks throughout the training process. This suggests that the model does need stability and better learning efficiency. In contrast, the ViT-based model Trans-Unet and Swin-Unet demonstrate low training losses for the entire training process, confirming efficient and effective learning of the models on the dataset. The hybrid model HTC-Net has a relatively high loss compared to the VM-UNet. Moreover, the least high and low peaks can be observed in the training loss curve of the proposed EDTNet, underscoring its superior learning efficiency and potential effectiveness in lung nodule segmentation.

### 4.6. Training and validation time evaluation

We evaluated the training time (hours) and validation time (minutes) of the Unet [41], ResUNet++ [42], U-NET 3+ [43], DeepLabV3+ [44], SegNet [45], Trans-Unet [46], Swin-UNet [32], and proposed EDTNet on DS1 shown in Table 4. The SegNet has 17.4x10^6^ parameters and it took least training and validation time. Further, Trans-Unet has highest trainable parameters 80x10^6^. The HTC-Net and VM-UNet has slightly high training and validation time compared to classical CNN based encoder and decoder models. The proposed EDTNet training and validation time is less then compared to its counterpart ViT based models Trans-Unet and Swin-UNet. Similarly, on DS2, the training and validation time of the CNN based models are relatively less compared to transformer-based model. Trans-Unet has highest training time of 172 minutes (m) on DS1 and 214(m) on DS2. The proposed EDTNet has 95(m) and 145(m) training time on DS1 and DS2, respectively.

**Table 4:** Comparison of the training and validation time on DS1.

	DS1			DS2	
Model	Train (m)	Val(s)	Train (m)	Val(s)	Parameters
Unet [41]	85	45	103	67	39.39x10^6^
ResUNet++[42]	103	57	137	86	35.7x10^6^
U-NET 3+ [43]	87	49	105	53	26.97x10^6^
DeepLabV3+ [44]	92	65	127	73	43.9x10^6^
SegNet [45]	72	39	93	48	17.4x10^6^
Trans-Unet [46]	172	71	214	172	80x10^6^
Swin-UNet [32]	126	67	176	127	36.6x10^6^
HTC-Net [39]	121	63	167	123	35.2x10^6^
VM-UNet [40]	102	56	127	83	34.7x10^6^
EDTNet	95	62	145	113	32.25x10^6^

### 4.7. Ablation study

In this section, we present the effect of different components on model performance. As we can see in Table 5, that the 1xESLA-MSA+PM (patch merging) +PE (positional encoding) combination has IoU values of 93.17% and 93.87%, respectively, on DS1 and DS2. Furthermore, the inclusion of the Conv_embedding improved the IoU value. We removed the PM and added the conv_embedding layer, which increases the IoU value by 0.85% and 0.88% on DS1 and DS2, respectively. Further, we added a 1xESGA-MSA block to provide global attention and found improvement in IoU on DS1 and DS2. The highest IoU value is achieved with components 2xESLA-MSA + 2xESGA-MSA +Conv_downsampling + Conv_embedding +Skip Attention. In addition, we found increasing the transformer layer increases the computation costs without a significant increase in the IoU value.

**Table 5:** Different components effects on model performance.

Components	IoU on DS1	IoU on DS2
1xESLA-MSA+PM+PE	93.17%	93.87%
1xESLA-MSA+PM+Conv_embedding	94.02%	94.28%
1xESLA-MSA+Conv_downsampling+Conv_embedding	94.87	95.16%
1xESLA-MSA+1xESGA-MSA+Conv_downsampling+ Conv_embedding	95.10%	95.67%
2xESLA-MSA+2xESGA-MSA+Conv_downsampling+ Conv_embedding+Skip Attention	95.81%	96.06%

### 4.8. Performance evaluation on the LIDC-IDRI dataset

The LIDC-IDRI is an open-source dataset containing 1018 CT-scan images collected from 1010 patients. Four physicians mark the category and location of the nodules for diagnosis of the instances in each image. The nodules range from very small (less than 3 mm) to larger ones (greater than 3 mm), and annotations are provided in XML format [59]. We selected the slices whose nodule size is > = 3mm and the lesions marked by the first and fourth radiologists. For the experiment, we selected 3597 images of size 512x512 pixels. After that, the centroid of each scan was obtained from the XML file, and images were cropped to 64x64 pixels to generate ROI (region of interest). Furthermore, mask images from ROI are generated using annotation defined in the dataset. The mask image and original image are fed to the model for training and validation under the same experimental condition defined in section 4.1, and performance metrics are calculated using the formula defined in section 4.2. The performance measures on the LIDC-IDRI dataset are shown in Table 6. We can see that the SegNet has precision and IoU values of 72.25% and 70.38%, Whereas Unet has 74.13% and 71.16% precision and IoU values. The ResUnet++ and U-NET3+ have SDC values of 72.67% and 71.47%, respectively. Furthermore, DeepLabV3+ and Trans-Unet have 75.40% and 78.62% IoU value. The HTC-Net and VM-Unet improved the performance measures and achieved 82.06% and 83.70% IoU values. Meanwhile, the proposed EDTNet has the highest IoU and SDC values, 85.12% and 84.25%, respectively.

**Table 6:** Performance matrices on the LIDC-IDRI dataset.

Model	Precision (%)	Sensitivity (%)	IoU(%)	SDC (%)
Unet [41]	74.13	72.54	71.16	70.63
ResUNet++ [42]	76.72	75.69	74.02	72.67
U-NET 3+ [43]	75.94	76.82	73.15	71.47
DeepLabV3+[44]	78.29	77.53	75.40	74.29
SegNet [45]	72.25	74.28	70.38	70.08
Trans-Unet [46]	79.72	81.30	78.62	77.92
Swin-UNet [32]	80.56	82.57	81.35	80.24
HTC-Net [39]	83.17	84.31	82.06	81.42
VM-UNet [40]	85.50	84.89	83.70	82.13
Proposed EDTNet	**87.96**	**86.75**	**85.12**	**84.25**

**Fig 6:**
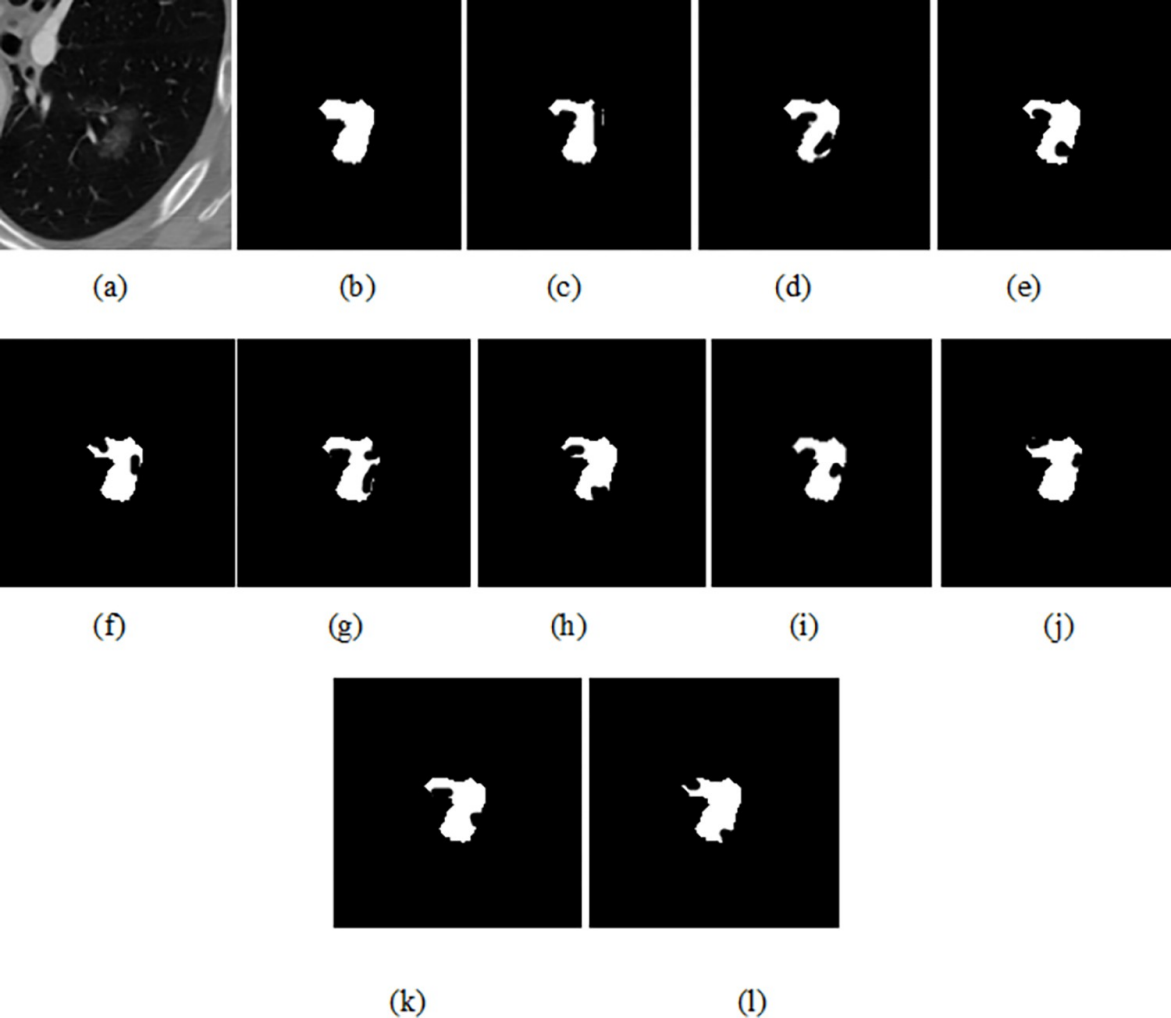
The predicted Figure, a) original image, (b) GT ((c)Unet (d) ResUNet++ (e) U-NET3+ (f) DeepLabV3+ (g)SegNet (h)Trans-Unet (i) Swin-UNet (j)HTC-Net, (k)VM-UNet and (l) EDTNet.

The visual results of the proposed EDTNet and Unet, ResUNet++, U-NET3+, DeepLabV3+, SegNet, Trans-Unet, Swin-UNet, HTC-Net and VM-UNet is shown in Fig 6. We can notice that the visual results of the Unet and SegNet have a high volume of noise, and original details are missing. The ResUNet++ and UNET3+ have better visual images. Moreover, Trans-Unet and Swin-Unet have finer lung nodule details, and less noise is present in the predicted image. The HTC-Net and VM-Unet visual results have less noise compared to other methods. In contrast, the EDTNet visual image is close to GT, and contains more details of the object.

### 4.9. Performance comparison with recent methods

We compared the performance of the proposed recent method, as shown in Table 7. Table 7 shows that the method proposed by Canayaz et al. [60] utilized LinkNet and achieved 70.56% DSC value. Meanwhile, the SAtUNet-based method obtained 81.80%. Furthermore, improvement in the DSC value can be noticed in the method [61]. The 3D convolution-based method S3DV-Net achieved 84.57% DSC value. At the same time, the Wavelet U-Net-based approach obtained a 93.60% DSC value. Moreover, the proposed EDTNet achieved a DSC value of 96.15% and 97.85% on the DS1 and DS2, respectively.

**Table 7:** Performance comparison with recent methods.

Method	Model	Dataset size	SDC
Canayaz et al. [60]	LinkNet	758 images	70.56%
Selvadass et al.[62]	SAtUNet	6787 images	81.80%
Wang et al. [61]	SaraNet	7397 images	89.11%
Xu et al. [63]	S3DV-Net	1186 images	84.57%
Agnes et al. [64]	Wavelet U-Net++	3000 images	93.60%
Proposed method	EDTNet	1224 images (DS1 1657 images (DS2)	96.15% 97.85%

### 4.10. Visual results on different sizes of lung nodules

**Fig 7:**
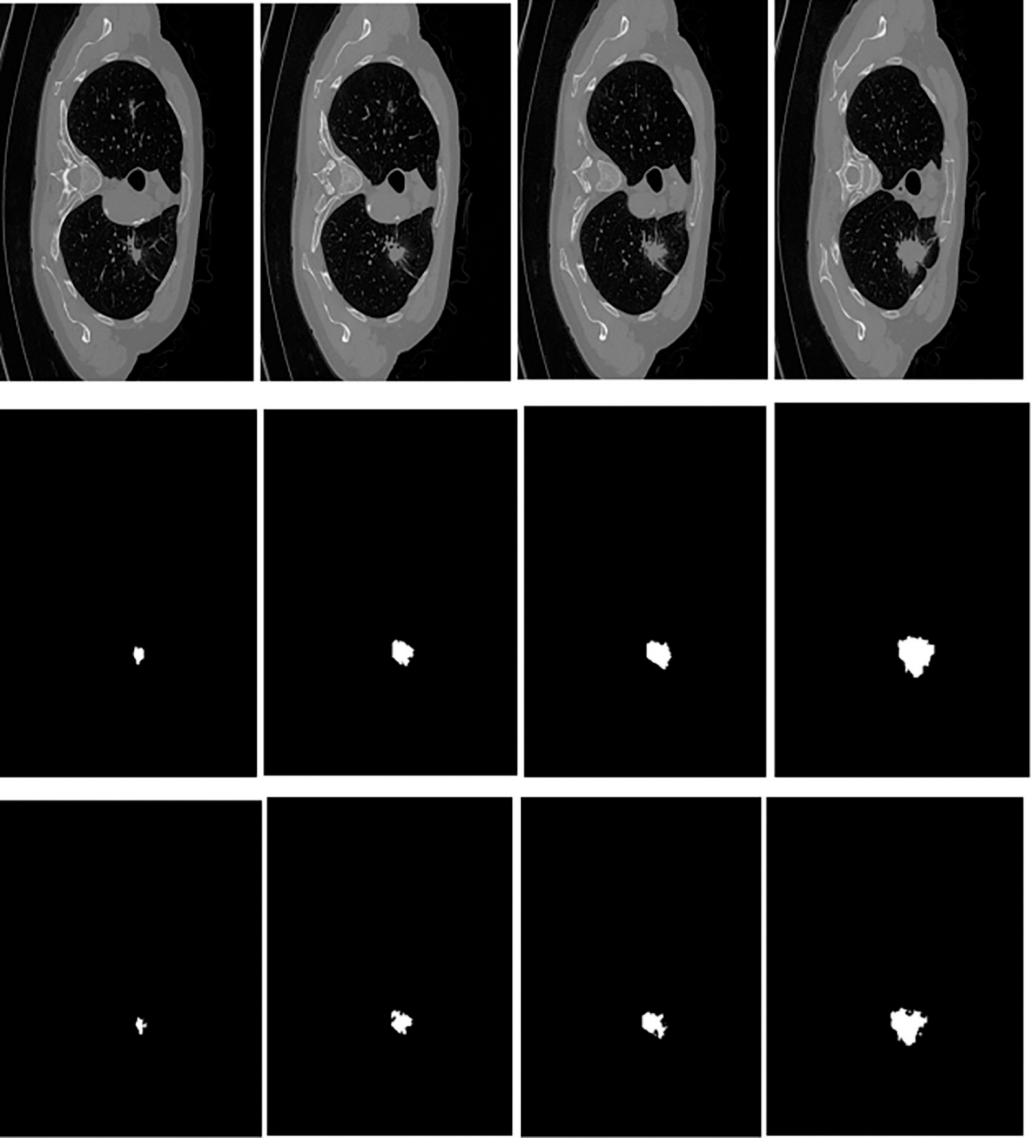
Visual results of the proposed model on different size nodules on DS2.

We selected the sample image of DS2 (Decathlon challenge) with varying lung nodules, shown in Fig 7. The top row contains original images, the middle row GT and the bottom row segmented results of the proposed EDTNet. We can observe that the model can localize small nodules with low noise. On the other hand, larger nodules’ visual results are very close to GTs with rich object details.

## 5. Conclusion

The proposed study developed EDTNet for PNS. The traditional CNN-based encoders and decoders cannot capture long-range spatial dependencies. Due to this, they have less optimal performance in complex object segmentation. Therefore, we designed a transformer-based model to address these challenges. Leveraging an enhanced spatial attention-based Vision Transformer (ViT) as both encoder and decoder, our EDTNet incorporates two successive transformer blocks and downsampling layers in the encoder, along with upsampling layers, transformer blocks, and a patch-expanding layer in the decoder. Our architecture includes a bottleneck with three global transformer blocks to enhance the receptive field and the decoder’s functionality. Furthermore, we incorporated skip connections to facilitate the symmetrical interaction between the encoder and decoder, enabling the retrieval of intricate details in the output. The EDTNet performance is compared with Unet, ResUNet++, U-NET 3+, DeepLabV3+, SegNet, Trans-Unet and Swin-UNet. The quantitative and visual results of the EDTNet are better than those of the other methods. The EDTNet precision, IOU, and DSC values on the DS1 are 96.27%, 95.81% and 96.15%, respectively. On the other hand, DS2 has IoU and sensitivity values of 96.06% and 98.84%, respectively.

The performance of the proposed method is evaluated on specific datasets, and its generalization to diverse datasets with varying characteristics needs to be evaluated. The transformer-based architecture introduces higher computational complexity compared to traditional CNN-based models. This could pose challenges in real-time applications. The intricate nature of transformer-based models might compromise the interpretability of the segmentation results. In future studies, the EDTNet will be tested on diverse datasets under various imaging conditions to establish the robustness of the proposed EDTNet. In addition, we will refine the model architecture to make the EDTNet more feasible for real-time PNS. Further, exploring ensemble-learning techniques by combining the strengths of the EDTNet with other state-of-the-art models might improve segmentation accuracy and robustness.
